# GEP100-Arf6-AMAP1-Cortactin Pathway Frequently Used in Cancer Invasion Is Activated by VEGFR2 to Promote Angiogenesis

**DOI:** 10.1371/journal.pone.0023359

**Published:** 2011-08-15

**Authors:** Ari Hashimoto, Shigeru Hashimoto, Ryo Ando, Kosuke Noda, Eiji Ogawa, Hirokazu Kotani, Mayumi Hirose, Toshi Menju, Masaki Morishige, Toshiaki Manabe, Yoshinobu Toda, Susumu Ishida, Hisataka Sabe

**Affiliations:** 1 Department of Molecular Biology, Hokkaido University Graduate School of Medicine, Sapporo, Japan; 2 Department of Molecular Biology, Osaka Bioscience Institute, Suita, Osaka, Japan; 3 Department of Ophthalmology, Hokkaido University Graduate School of Medicine, Sapporo, Japan; 4 Laboratory of Diagnostic Pathology, Faculty of Medicine, Kyoto University, Kyoto, Japan; 5 Department of Thoracic Surgery, Faculty of Medicine, Kyoto University, Kyoto, Japan; 6 Center for Anatomical Studies, Kyoto University Graduate School of Medicine, Kyoto, Japan; 7 Laboratory of Supramolecular Crystallography, Institute for Protein Research, Osaka University, Osaka, Japan; 8 Department of Neurosurgery, School of Medicine, Oita University, Oita, Japan; University of Chicago, United States of America

## Abstract

Angiogenesis and cancer invasiveness greatly contribute to cancer malignancy.

Arf6 and its effector, AMAP1, are frequently overexpressed in breast cancer, and constitute a central pathway to induce the invasion and metastasis. In this pathway, Arf6 is activated by EGFR via GEP100. Arf6 is highly expressed also in human umbilical vein endothelial cells (HUVECs) and is implicated in angiogenesis. Here, we found that HUVECs also highly express AMAP1, and that vascular endothelial growth factor receptor-2 (VEGFR2) recruits GEP100 to activate Arf6. AMAP1 functions by binding to cortactin in cancer invasion and metastasis. We demonstrate that the same GEP100-Arf6-AMAP1-cortactin pathway is essential for angiogenesis activities, including cell migration and tubular formation, as well as for the enhancement of cell permeability and VE-cadherin endocytosis of VEGF-stimulated HUVECs. Components of this pathway are highly expressed in pathologic angiogenesis, and blocking of this pathway effectively inhibits VEGF- or tumor-induced angiogenesis and choroidal neovascularization. The GEP100-Arf6-AMAP1-cortactin pathway, activated by receptor tyrosine kinases, appears to be common in angiogenesis and cancer invasion and metastasis, and provides their new therapeutic targets.

## Introduction

Vascular endothelial growth factors (VEGFs) are major factors involved in angiogenesis [1]–[4]. A family member of VEGFs, namely VEGF-A, was originally discovered as a vascular-permeability factor [5]; and the primary function of VEGF signaling involves enhancement of endothelial-cell permeability and vascular leakage [6]. A small GTPase, Arf6, has been implicated in VEGF signaling and angiogenesis. It has been shown that expression of a dominant-negative form of Arf6, Arf6 (T27N), in human umbilical vein endothelial cells (HUVECs) inhibits vascular endothelial growth factor receptor-2 (VEGFR2)-mediated intracellular signaling, such as Rac1 activation [7]. Consistently, suppression of Arf6 activity via the Slit2-Robo4 pathway blocks angiogenesis and promotes vascular stability [8]. However, the mechanism as to how VEGFR2 regulates Arf6 activity, as well as the mechanisms by which Arf6 functions in angiogenesis, and also in other aspects of VEGF signaling, still largely remains elusive.

A small GTPase, Arf6, primarily regulates the recycling of plasma membrane components and plays pleiotropic roles, including membrane protrusion and remodeling [9], [10]. We have shown previously that different breast cancer cells overexpress both Arf6 and its effector, AMAP1; and that overexpressed Arf6 and AMAP1 then constitute a robust signaling axis to induce invasion and metastasis [11]–[15]. In invasion and metastasis, GEP100, a guanine nucleotide exchanger for Arf GTPases, is primarily responsible for Arf6 activation, and this activation requires the association of GEP100 with ligand-activated epidermal growth factor receptor (EGFR) [15]. Pathological analyses revealed that components of this pathway are highly expressed in 40–80% of primary tumors of the human breast [12], [15]. AMAP1 functions by binding to cortactin in cancer invasion and metastasis. Blocking of the GEP100-Arf6-AMAP1-cortactin pathway by siRNAs or inhibitors effectively blocks breast cancer invasion and metastasis [11]–[13], [15]. Read-outs of this Arf6 pathway include the disruption of E-cadherin-based cell-cell adhesions [15], by inducing E-cadherin endocytosis (our unpublished results).

Protein expression of Arf6 is markedly augmented in HUVECs when cultured with VEGF, and in a mouse hindlimb ischemia model in which angiogenesis is primarily dependent on VEGF [7], [16]. Here, we found that HUVECs also highly express AMAP1, comparable to the levels observed in highly invasive breast cancer cells. We also found that GEP100 physically associates with ligand-activated VEGFR2 to activate Arf6, and that Arf6 then recruits AMAP1. Like cancer invasion and metastasis, AMAP1 functions by binding to cortactin in angiogenesis. This GEP100-Arf6-AMAP1-cortactin pathway is essential not only for VEGF-induced endothelial cell migration and tubular formation, but also for VEGF-induced enhancement of VE-cadherin endocytosis and cell permeability. Components of this pathway are highly expressed in CD31-positive vessels with pathologic angiogenesis, and blocking of this pathway effectively inhibits pathologic angiogenesis. Our results reveal that the GEP100-Arf6-AMAP1-cortactin pathway, activated by growth factor receptor tyrosine kinases, is common in angiogenesis and invasion and metastasis of some breast cancer cells, and hence provides new therapeutic targets for human disorders characterized by hyper-angiogenesis and malignant cancer development.

## Materials and Methods

### Cells

HUVECs were purchased from Iwaki and grown in endothelial growth medium-2 (EGM2; Lonza), according to the manufacturer's instruction. Note that EGM2 contains a low concentration of VEGF, a concentration which is not open to public. MDA-MB-231 and MCF7 cells, obtained from the American Type Culture Collection, were cultured as described previously [15]. Cos-7 cells were cultured in DMEM with 10% fetal calf serum (FCS, Hyclone).

### Angiogenesis

Quantification of angiogenic responses was performed by the directed *in vivo* angiogenesis assay (DIVAA, Trevigen), according to the manufacturer's instructions. Briefly, 20 µl of VEGF (500 ng ml^−1^) or MDA-MB-231 cells (5×10^6^ ml^−1^) was injected into angioreactor tubes, which were filled with basement membrane extracts; and tubes were then implanted subcutaneously into the dorsal areas of nude mice. Nine days later the tubes were collected and the amounts of Isolectin B4 accumulated within the basement membrane extracts were measured using a multiplate fluorescent reader (ARVO, Perkin Elmer), after proteolytic digestion of the basement membrane. For siRNA treatment, RNA duplexes were mixed with AteloGene (Koken), according to the manufacturer's instructions; and 200 µl of the mixture was injected into the bottom sides of angioreactor tubes implanted in mice, at day 0 and day 4. P4-TAT peptide and the control SC peptide were added into the angioreactor tubes before implantation. These studies were performed at Osaka Bioscience Institute, and the protocols used for animal experiments in this study were approved by the Animal Research Committee of Osaka Bioscience Institute (Permit number: 07-103).

### Laser-induced CNV

CNV was conducted as described previously [17], [18]. One day before the laser administration, 5 mg kg^−1^ P4-TAT or SC peptide was injected intraperitoneally into 2-month-old male C57BL/6 mice (CLEA Japan). The next day mice were anesthetized with pentobarbital (0.05 mg g^−1^ body weight) and their pupils dilated with 0.5% phenylephrine and 0.5% tropicamide (Santen). CNV was induced with a 532 nm laser (Lumenis Novus Spectra). Four laser spots (200 mW power, 75 µm spot size, 100 msec) were placed in each eye using a slit-lamp delivery system and a cover glass as a contact lens. Production of a bubble at the time of laser treatment, indicating rupture of the Bruch's membrane, is an important factor in obtaining CNV; therefore, only burns in which a bubble was produced were included in this study. Immediately after laser treatment, 5 mg kg^−1^ P4-TAT or control scrambled peptide was injected intraperitoneally every day for 7 days. On experimental day 8, mice were anesthetized and perfused through the left ventricle with 5 ml PBS followed by 2 ml of 0.5% FITC-labeled dextran (Mr 2,000 kDa, Sigma) in 1% gelatin. The eyes were enucleated and fixed in 2% paraformaldehyde for 30 min. The anterior segment and retina were then removed from the eyecup. About 4 to 6 relaxing radial incisions were made, and the remaining retinal pigment epithelium (RPE)-choroidal-scleral complex was flatmounted with Vectashield Mounting Medium (Dako) and coverslipped. Flatmounts were examined with a microscope (BIOREVO; Keyence) and images of each CNV were digitally stored. Eyes with hemorrhagic complications such as vitreous hemorrhage or subretinal hemorrhage caused by laser irradiation were excluded from the evaluation. The average size of the CNV lesions was then measured, and data are presented as the mean ± s.e.m. with *n* as indicated. The animal experiments were conducted in accordance with the Association for Research in Vision and Ophthalmology Statement for the Use of Animals in Ophthalmic and Vision Research and the Committee for Animal Use and Care of Hokkaido University.

### VE-cadherin endocytosis

VE-cadherin endocytosis was assayed by the VE-cadherin antibody-binding method, as described [19]. Briefly, HUVECs, plated at a confluent density, were prestarved with EGM2 without serum for 4 h, and then incubating with a monoclonal antibody BV6 (10 mg ml^−1^), which is against the extracellular domain of VE-cadherin, at 4°C for 1 h. After washing off the unbound antibody by rinsing cells with ice-cold EGM2, cells were then incubated at 37°C for 30 min in the presence or absence of VEGF (50 ng ml^−1^). Cells were then washed with 25 mM glycine (pH 2.5) containing 3% bovine serum albumin for 15 min at an ambient temperature to remove surface-bound antibodies, and fixed in 4% paraformaldehyde, permeabilized with 0.5% Triton X-100, and subjected to labeling of internalized BV6 molecules by use of an antibody against mouse IgG conjugated with Alexa Fluor 546 (Molecular Probes). For siRNA treatment, cells were preincubated with RNA duplexes for 48 h, before plating. For TAT peptide treatment, cells were incubated with TAT peptides for 30 min at 37°C between the 4 h starvation and the labeling with BV6. The number of cells exhibiting at least one group of five or more acid-resistant VE-cadherin-positive vesicle-like structures was counted (n>300). Results are expressed as the mean ± s.e.m. of three independent experiments, and statical analysis was performed using ANOVA.

### Other methods

GST-GGA pulldown, immunoprecipitation, immunoblotting, antibodies and chemicals, cDNAs, siRNAs, transfections, tubular formation, chemotactic transwell migration, two dimentional cell migration activity immunohistochemical staining, cell permeability, immunofluorescent microscopy, GST-PH binding, viability and RT-PCR are described in the Supporting Materials and Methods S1.

## Results

### VEGF activates Arf6 via GEP100 in endothelial cells

**Figure 1:**
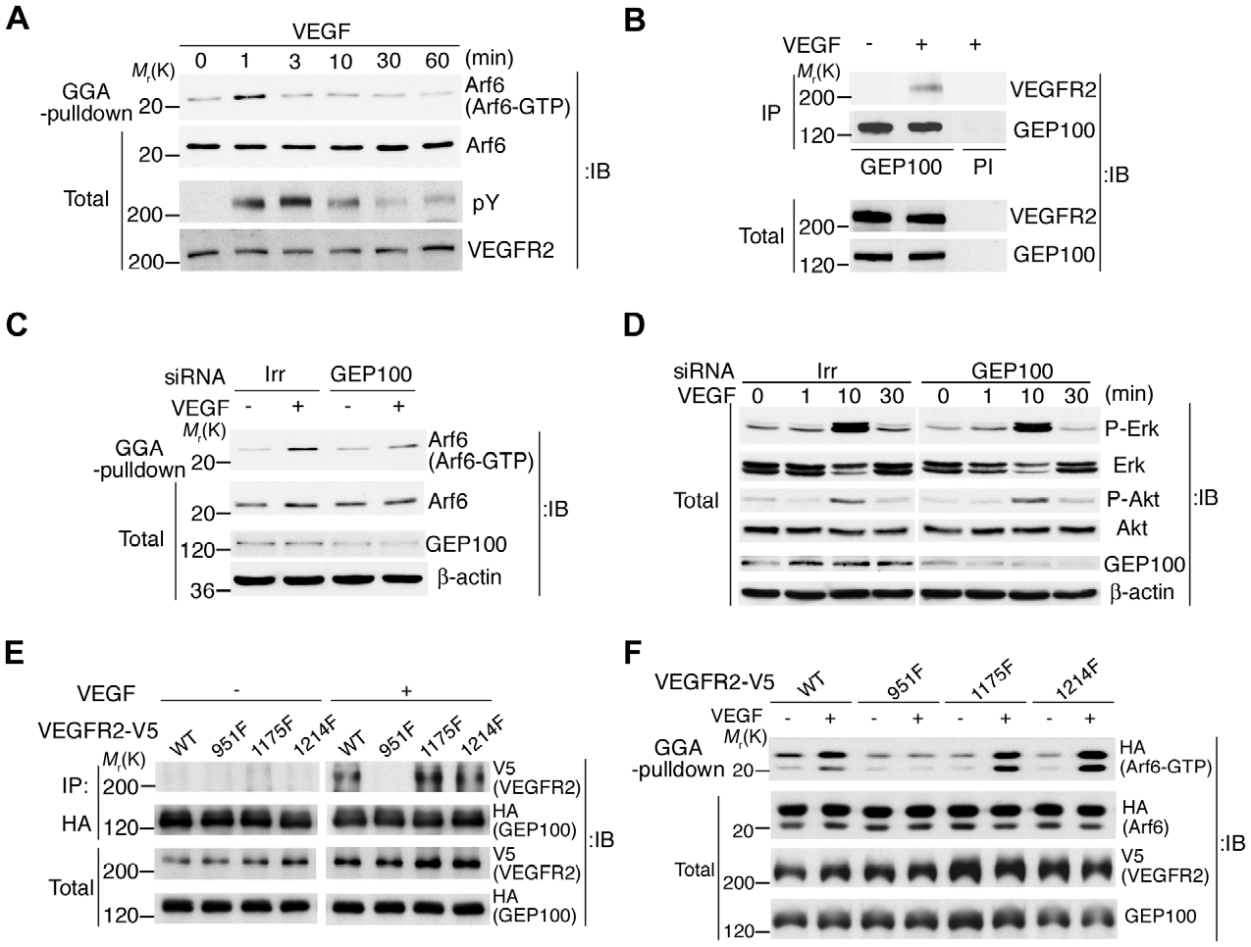
GEP100 associates with ligand-activated VEGFR2 to activate Arf6. **A**, Activation of Arf6 and tyrosine phosphorylation of VEGFR2 upon VEGF stimulation of HUVECs. **B**, Coprecipitation of GEP100 with VEGFR2 upon VEGF stimulation of HUVECs, analysed by anti-GEP100 immunoprecipitation (IP) and anti-VEGFR2 immunoblot. PI, pre-immune serum. **C–D**, Activities of Arf6 (**C**), and Erk and Akt (**D**) in HUVECs transfected with siRNAs for GEP100 or irrelevant sequences (Irr) upon VEGF stimulation. **E**, Coprecipitation of VEGFR2-V5 and its tyrosine phosphorylation-deficient mutants (951F, 1175F and 1214F) with HA-GEP100 expressed in Cos-7 cells, analysed by anti-HA (GEP100) immunoprecipitation and anti-V5 (VEGFR2) immunoblot. **F**, Activation of Arf6-HA by VEGFR2-V5 or its mutants (951F, 1175F and 1214F) upon VEGF stimulation in Cos-7 cells, in which non-tagged GEP100 cDNA was simultaneously transfected. In **A**–**F**, 10 ng ml^−1^ VEGF was used for stimulation for 1 min (+) or for the indicated times, while controls included cells without stimulation (0 min or −). Arf6 activities were assessed by the GST-GGA pulldown method (**A**, **C**, **F**). Total, total cell lysates (20 µg). In **A–D**, HUVECs were cultured in low serum (0.5% FCS) medium for 16 h before stimulation. These assays were performed at least twice and representative figures are shown.

Although Arf6 activity is implicated in VEGF signaling and angiogenesis, it is not yet shown whether VEGF activates Arf6. We found that stimulation of primary culture of HUVECs by VEGF indeed induced activation of endogenous Arf6 (Figure 1A). This activation was transient, peaked at 1 min and then declined, as observed with other small GTPases [20]. HUVECs express predominantly VEGFR2 among the VEGFR-family members. Tyrosine phosphorylation of VEGFR2 occurred within 1 min, peaked at 3 min and declined by 10 min after the stimulation (Figure 1A).

Although signaling pathways downstream of VEGFR2 have been extensively studied [21], none of them could interpret the mechanisms of Arf6 activation. Moreover, more than a single type of GEF can activate Arf6 [22], [23]. We then sought to identify GEF(s) primarily responsible for this VEGF-induced Arf6 activation. GEP100 binds to tyrosine phosphorylated EGFR [15]. We tested whether GEP100 also binds to tyrosine phosphorylated VEGFR2, and found that VEGFR2 is coprecipitated with GEP100 in HUVECs endogenously, when cells were stimulated by VEGF (Figure 1B). We then knocked down GEP100 by the siRNA method in HUVECs, and found that this knockdown largely abolished the VEGF-induced activation of Arf6 (Figure 1C and Figure S4). These results suggest that GEP100 is primarily responsible for the VEGF-induced activation of Arf6 in HUVECs.

VEGF signaling is known to activate Erk and Akt [21]. Silencing of GEP100 does not affect the activation of Erk and Akt in HUVECs (Figure 1D). These results, together with the results described above, confirm the specificity of GEP100 in VEGF signaling, and indicate that the VEGF signaling pathway that activates Arf6 is independent of that activating Erk and Akt in HUVECs. Moreover, activation of Erk and Akt both occur 10 min after the VEGF stimulation, which is much slower than the activation of Arf6.

### Mode of GEP100 binding to ligand-activated VEGFR2

We next investigated the precise mechanism by which ligand-activated VEGFR2 employs GEP100. The PH domain of GEP100 binds to certain phosphorylated tyrosines of EGFR [15]. We first examined whether this PH domain also binds to phosphorylated tyrosines of VEGFR2. For this, we expressed V5-tagged VEGFR2 in Cos-7 cells, and their lysates were pulled-down *in vitro* with the PH domain of GEP100, fused to glutathione-*s*-transferase (GST). We found that the GST-GEP100 PH domain pulls down ligand-activated VEGFR2-V5, while PH domains of ARNO or phospholipase Cδ do not (Figure S1). VEGFR2 has 6 major tyrosines, phosphorylated upon VEGF stimulation: Tyr951, Tyr996, Tyr1054, Tyr1059, Tyr1175 and Tyr1214 [21] (see Figure S2). We synthesized these tyrosine peptides in their phosphorylated form, and found that the GST-GEP100 PH domain binds to the phosphorylated Tyr951 peptide, but not to the other phosphorylated peptides (Figure S3). Furthermore, the GST-GEP100 PH domain did not bind to the non-phosphorylated Tyr951 peptide (Figure S3). We confirmed phosphorylation of Tyr951 upon VEGF stimulation in HUVECs (Figure S5).

Based on these results, we then generated a mutant form of VEGFR2-V5, in which Tyr951 was changed to phenylalanine (951F) and expressed it in Cos-7 cells together with hemagglutinin (HA)-tagged GEP100, and found that this mutant is not coprecipitated with HA-GEP100 (Figure 1E). As a control, we confirmed that wild type VEGFR2-V5 is coprecipitated with HA-GEP100 (Figure 1E). Moreover, mutations of the other tyrosines, such as Tyr1175 and Tyr1214, into phenylalanine (1175F and 1214F, respectively) did not affect the coprecipitation (Figure 1E). We then expressed VEGFR2-V5 or its mutants together with Arf6-HA and GEP100 in Cos7 cells, and measured activities of Arf6-HA by use of the GST-GGA pulldown method [24]. We found that the 951F mutant of VEGFR2-V5 does not induce the activation of Arf6-HA in response to VEGF, while the 1175F and 1214F mutants, as well as wild type VEGFR2-V5, induce Arf6-HA activation (Figure 1F). These results indicate that VEGFR2 physically associates with GEP100 to activate Arf6 upon VEGF stimulation: an association which requires the binding of phosphorylated Tyr951 of VEGFR2 and the PH domain of GEP100. We also confirmed that the Tyr951-phosphorylated form of VEGFR2 is coprecipitated with GEP100 from HUVECs endogenously, upon VEGF stimulation (Figure S2).

### Requirement for Arf6 in VEGF-induced tubular formation and migration

**Figure 2:**
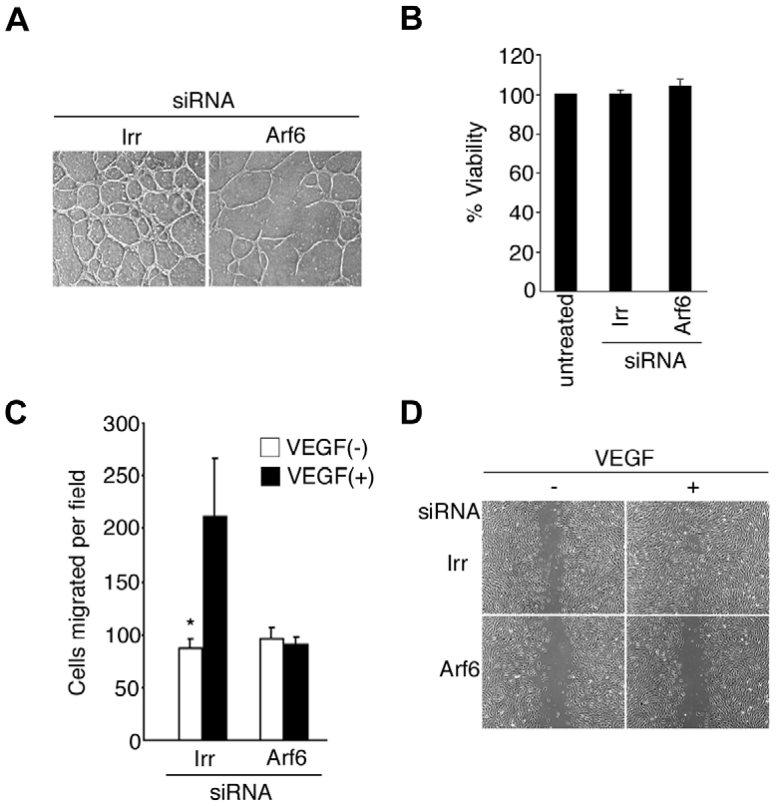
Arf6 is necessary for VEGF-induced angiogenic activities. HUVECs, treated with siRNAs for Arf6 or irrelevant sequences (Irr), were subjected to the tubular network formation assay in the presence of 10 ng ml^−1^ VEGF (**A**), to a viability assay (**B**), and to a migration assay using a modified Boyden chamber (**C**) or using a wound healing assay (**D**) in the presence and absence of VEGF (10 ng ml^−1^). In **A** and **D**, assays were performed more than two times, and representative figures are shown. In **B**, more than 1×10^4^ cells were scored in each assay. In **C**, data are presented as the number of cells observed per microscopic field (×20) which transmigrated the Boyden chamber filter. Six fields were counted in each assay. Error bars show mean ± s.e.m., n = 3. * p<0.05.

Tubular (or capillary-like) network formation of HUVECs cultured *in vitro* is one of the hallmark processes necessary for angiogenesis [1], [2]. To examine the involvement of Arf6 in VEGF-induced angiogenesis, we then tested the effects of Arf6 siRNA. Knockdown of Arf6 significantly impaired VEGF-induced tubular formation, as compared to control irrelevant RNA duplexes (Irr) (Figure 2A and Figure S4), without affecting cell viability (Figure 2B). VEGF-induced cell migratory activity is another hallmark of angiogenic activity [25]. Arf6 siRNA treatment abolished VEGF-induced trans-migration activities almost completely, which were assessed using modified Boyden chambers [26] (see Figure 2C). VEGF-induced two-dimensional migration activities, assessed by the wound healing assay [27], was also almost completely blocked by Arf6 siRNA treatment (Figure 2D).

### High levels of AMAP1 expression in HUVECs and involvement of GEP100 and AMAP1 in VEGF-induced angiogenic activities

**Figure 3:**
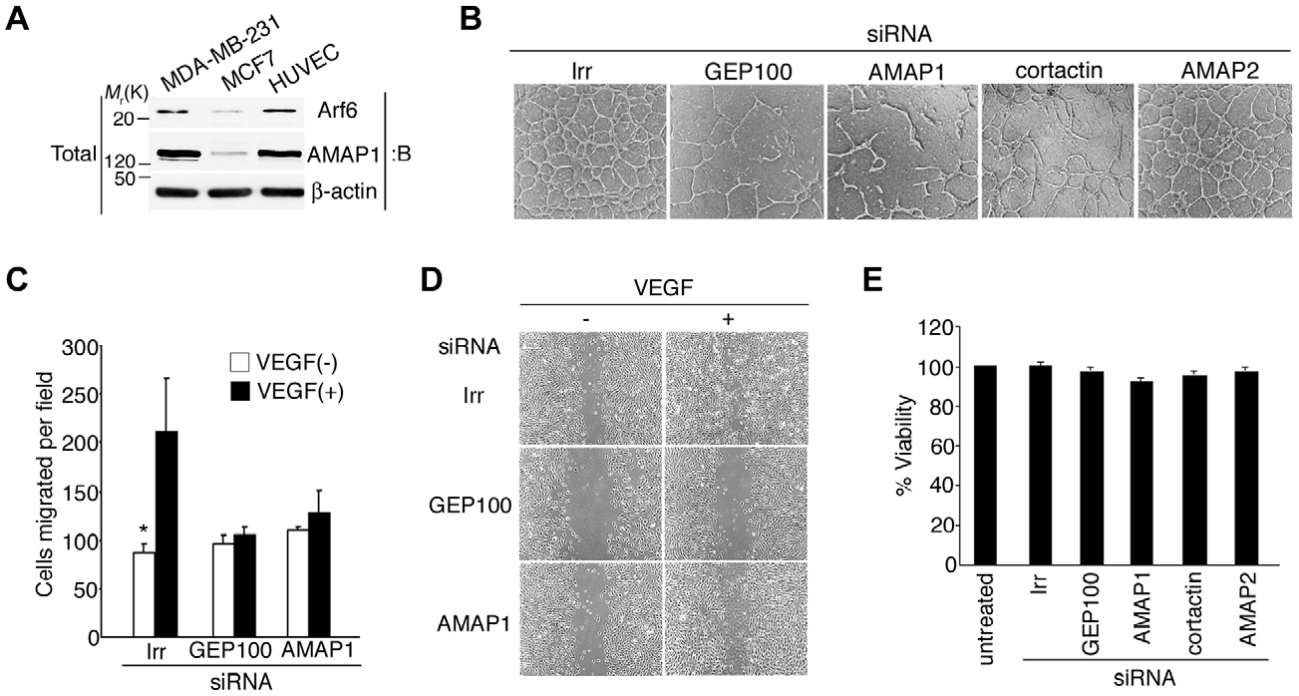
Arf6 signaling components, GEP100, AMAP1 and cortactin, are all necessary for VEGF-induced angiogenic activities. **A**, Expression of Arf6 and AMAP1 proteins in HUVECs and its comparison with those in invasive (MDA-MB-231) and non-invasive (MCF7) breast cancer cells, by immunoblotting 20 µg of total cell lysates using antibodies as indicated. β-actin was used as a control. **B**–**E**, HUVECs, treated with siRNAs for GEP100, AMAP1, cortactin or irrelevant sequences (Irr), were subjected to the tubular formation assay (**B**), modified Boyden chamber assay (**C**), wound healing assay (**D**) or cell viability assay (**E**), as in Figure 2. AMAP2 siRNAs were included as another control (**B**, **E**). These assays were performed at least two times, and representative figures are shown. Error bars show mean ± s.e.m., n = 3. * p<0.05.

AMAP1 is a downstream effector for Arf6, and functions in cancer invasion and metastasis [12]. Most malignant breast cancer cells with high invasive activities abnormally overexpress both Arf6 and AMAP1 proteins, while weakly- or non-invasive breast cancer cells express only marginal levels of these two proteins [11], [12]. HUVECs are known to express Arf6 at a high level [7], which we found to be almost equivalent to that observed with highly invasive MDA-MB-231 breast cancer cells (Figure 3A). HUVECs also express AMAP1 at a very high level, which is also comparable to MDA-MB-231 cells (Figure 3A).

We next examined whether GEP100 and AMAP1 are involved in VEGF-induced angiogenic activities *in vitro*. Knockdown of GEP100 and AMAP1 each significantly affected VEGF-induced tubular formation, and almost completely blocked VEGF-induced cell migratory activities (Figure 3B–3D and Figure S4), without affecting cell viability (Figure 3E). As a control, we also knocked down AMAP2 [28] (see Figure S4), a close isoform of AMAP1, and did not observe an inhibitory effect on tubular formation (Figure 3B).

### Requirement for cortactin and its association with AMAP1 in VEGF-induced angiogenic activities

**Figure 4:**
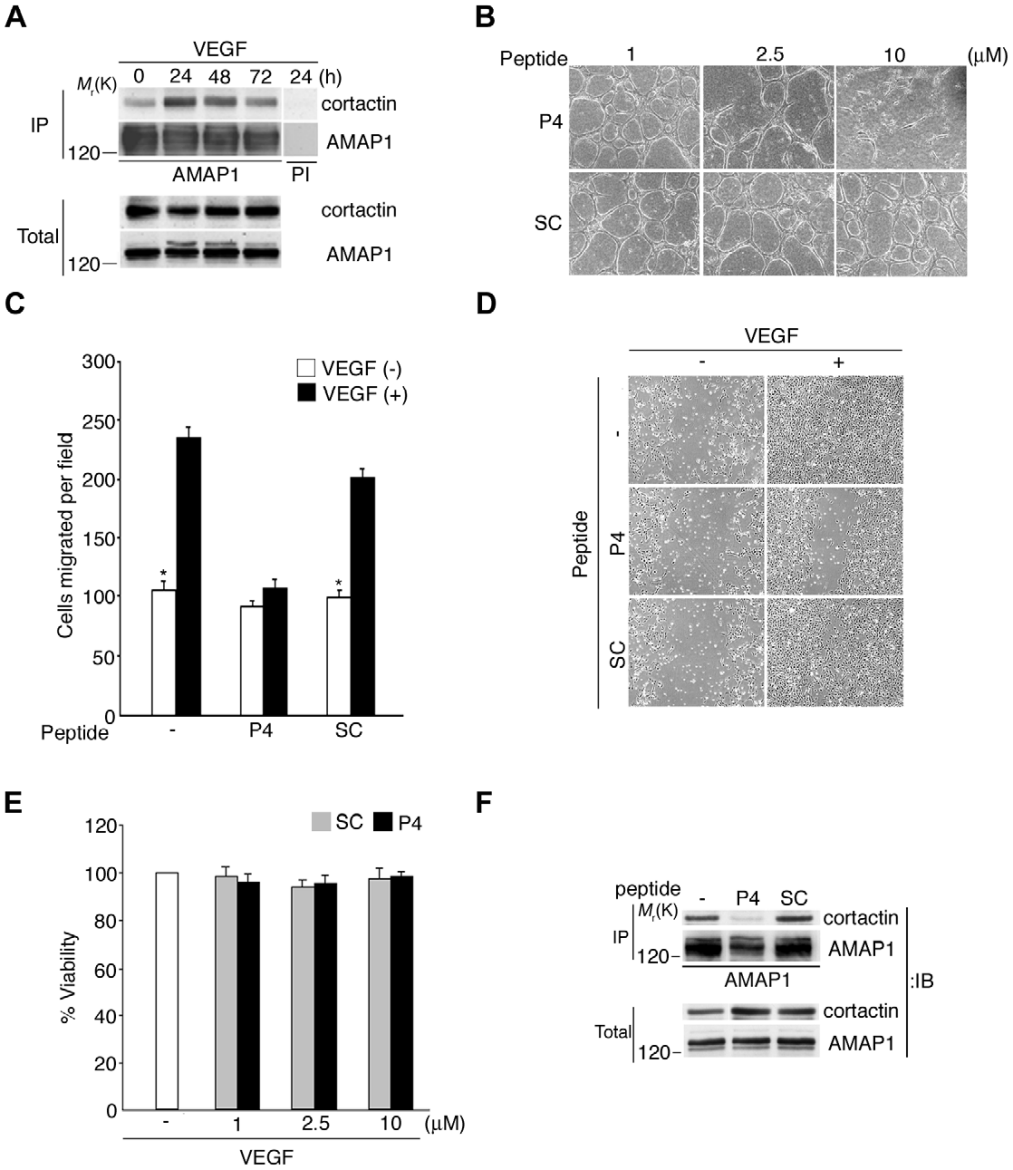
P4-TAT blocks VEGF-induced angiogenic activities. **A**, Coprecipitation of cortactin with AMAP1 in HUVECs cultured in the presence of 10 ng ml^−1^ VEGF, analysed by anti-AMAP1 immunoprecipitation and anti-cortactin immunoblot, as indicated. PI, pre-immune serum. **B**–**D**, HUVECs, cultured in the presence of P4-TAT (P4) or a scrambled cell permeable peptide (SC) at 10 µM (**C**, **D**, **F**) or at concentrations as indicated (**B**, **E**) for 1 h prior to analysis, were subjected to the tubular formation assay (**B**), modified Boyden chamber assay (**C**), wound healing assay (**D**) and cell viability assay (**E**), as in Figure 2, in the presence of the peptides. Coprecipitation of cortactin with AMAP1 in these cells was analysed as above (**F**). Total, total cell lysates (20 µg). These assays were performed at least two times, and representative figures are shown. Error bars show mean ± s.e.m., n = 3. * p<0.05.

AMAP1 functions by forming a complex with cortactin in invasive breast cancer cells [12], [13]. We found that AMAP1 forms a complex with cortactin also in HUVECs, and this complex formation significantly increased when cells were cultured with VEGF (Figure 4A). Moreover, cortactin siRNAs effectively inhibited VEGF-induced angiogenic activities *in vitro*, without affecting cell viability (Figure 3B and 3E).

We previously designed a cell-permeable peptide, namely P4-TAT, that blocks AMAP1 and cortactin binding, and hence inhibits cancer invasion and metastasis [13]. P4-TAT, but not the control scrambled TAT-peptide (SC), blocked VEGF-induced angiogenic activities *in vitro*, like tubular formation and cell migration, in a dose-dependent manner, without affecting cell viability (Figure 4B–4E). We confirmed that P4-TAT, but not SC, blocks endogenous binding of AMAP1 with cortactin in HUVECs (Figure 4F). These results indicate that AMAP1 functions via its complex formation with cortactin in HUVECs, and this complex formation is necessary for the VEGF-induced angiogenic activities.

### Involvement of the Arf6 signaling pathway in angiogenesis

**Figure 5:**
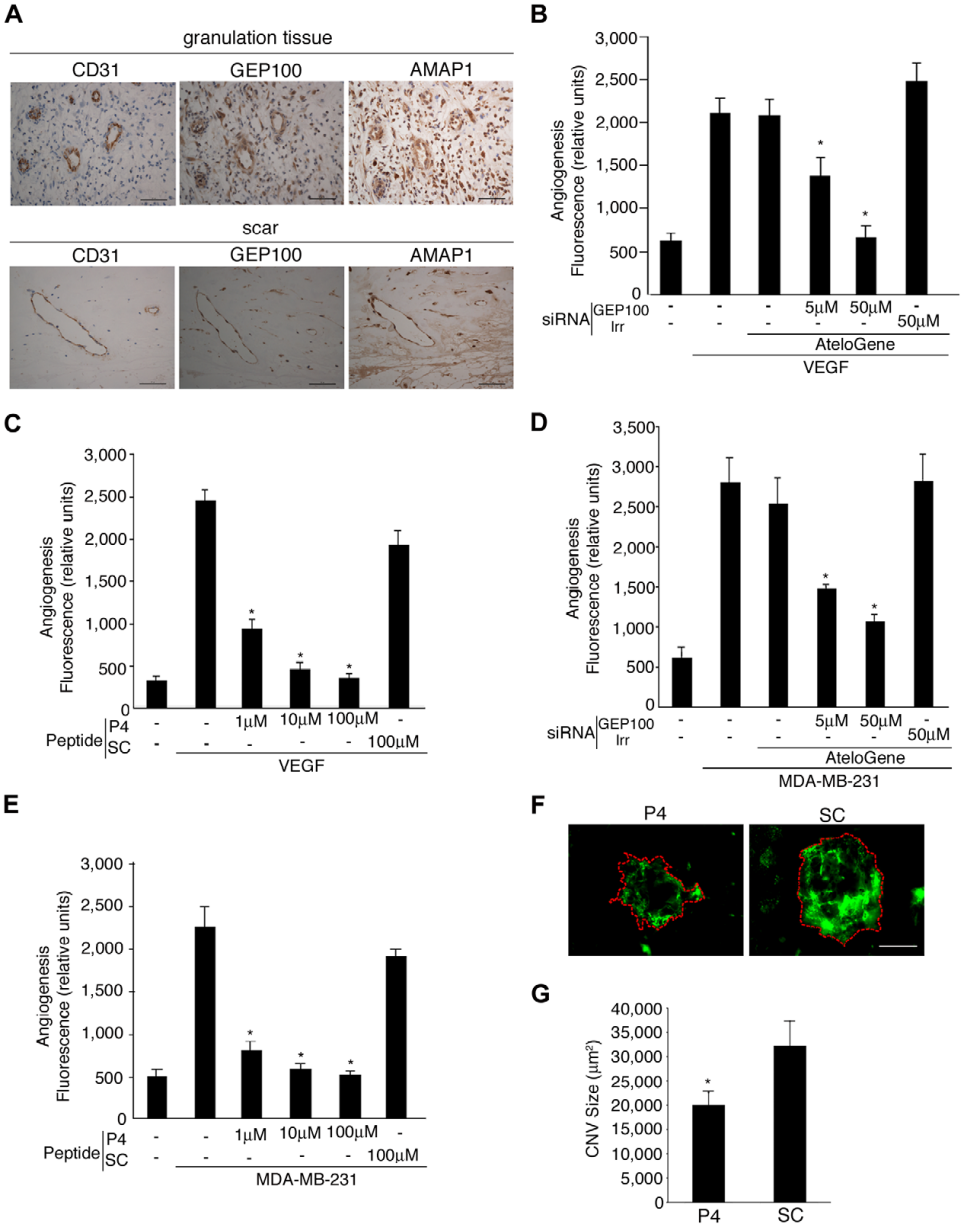
Blockage of pathologic angiogenesis by GEP100 siRNA and P4-TAT. **A**, Immunohistochemistry of granulation tissue and scar tissue sections by use of the indicated antibodies. Bars, 100 µm. **B**–**E**, Effects of GEP100 siRNAs and P4-TAT (P4) on angiogenesis were measured using angioreactors implanted into nude mice, that contained basement membrane extracts and VEGF (500 ng ml^−1^) or MDA-MB-231 cells (1×10^5^ cells); and amounts of Isolectin B4 accumulated within the basement membrane extracts were measured after incubation for 9 days. siRNAs, mixed with AteloGene at concentrations as indicated, were injected into mice at day 0 and day 4. TAT-peptides were added into angioreactors before implantation, at the indicated concentrations. An irrelevant RNA duplex (Irr) or a scrambled peptide (SC) was used as controls. Error bars show mean ± s.e.m., n = 8. * p<0.05. **F–G**, Effect of P4-TAT on CNV formation. Representative micrographs of CNV lesions in choroidal flatmounts from an animal treated with P4-TAT or SC. Red dashed line shows the extent of the CNV lesions filled with FITC-dextran. Scale bar, 100 µm. Quantitative analysis of the average CNV size is shown in **G**. Error bars show mean ± s.e.m., n = 70 to 77. *P<0.05.

We then examined whether the GEP100-Arf6-AMAP1-cortactin pathway is involved in pathologic angiogenesis *in vivo*. For this, we first confirmed that CD31-positive pathologic vessels [16] are strongly positive for GEP100 and AMAP1 (Figure 5A). Antibodies against Arf6 applicable to immunohistochemistry were not available. We then tested GEP100 siRNAs and P4-TAT. For this, angioreactor tubes [29], loaded with VEGF or MDA-MB-231 cells, were implanted into nude mice, and 9 days later the amounts of Isolectin B4 accumulated within the tubes were measured. MDA-MB-231 cells are known to produce several angiogenic factors [30]. Administration of GEP100 siRNAs, pre-mixed with AteloGene [31], into mice implanted with these angioreactors inhibited their angiogenesis in a dose-dependent manner, while an irrelevant RNA duplex did not (Figure 5B and 5D). P4-TAT, but not SC, also inhibited this angiogenesis in a dose-dependent manner (Figure 5C and 5E).

We moreover examined the effects of P4-TAT on choroidal neovascularization (CNV), which is the main cause of severe vision loss in patients with age-related macular degeneration [32], by the use of laser-induced choroidal neovascularization in mice [17], [18]. P4-TAT or SC peptide was injected intra-peritoneally into mice daily from a day before the laser treatment until the end of the experiment. We chose intra-peritoneal administration rather than direct injection into the eyes, to prevent injuring the eyes by the latter method. P4-TAT was also effective in inhibiting CNV (Figure 5F and 5G).

### Involvement of the Arf6 signaling pathway in endothelial permeability and VE-cadherin endocytosis

The primary function of VEGF signaling involves promoting endothelial cell permeability and vascular leakage. VEGF signaling induces endocytosis of VE-cadherin, and this endocytosis is crucial for the enhancement of endothelial permeability [33], [34].

**Figure 6:**
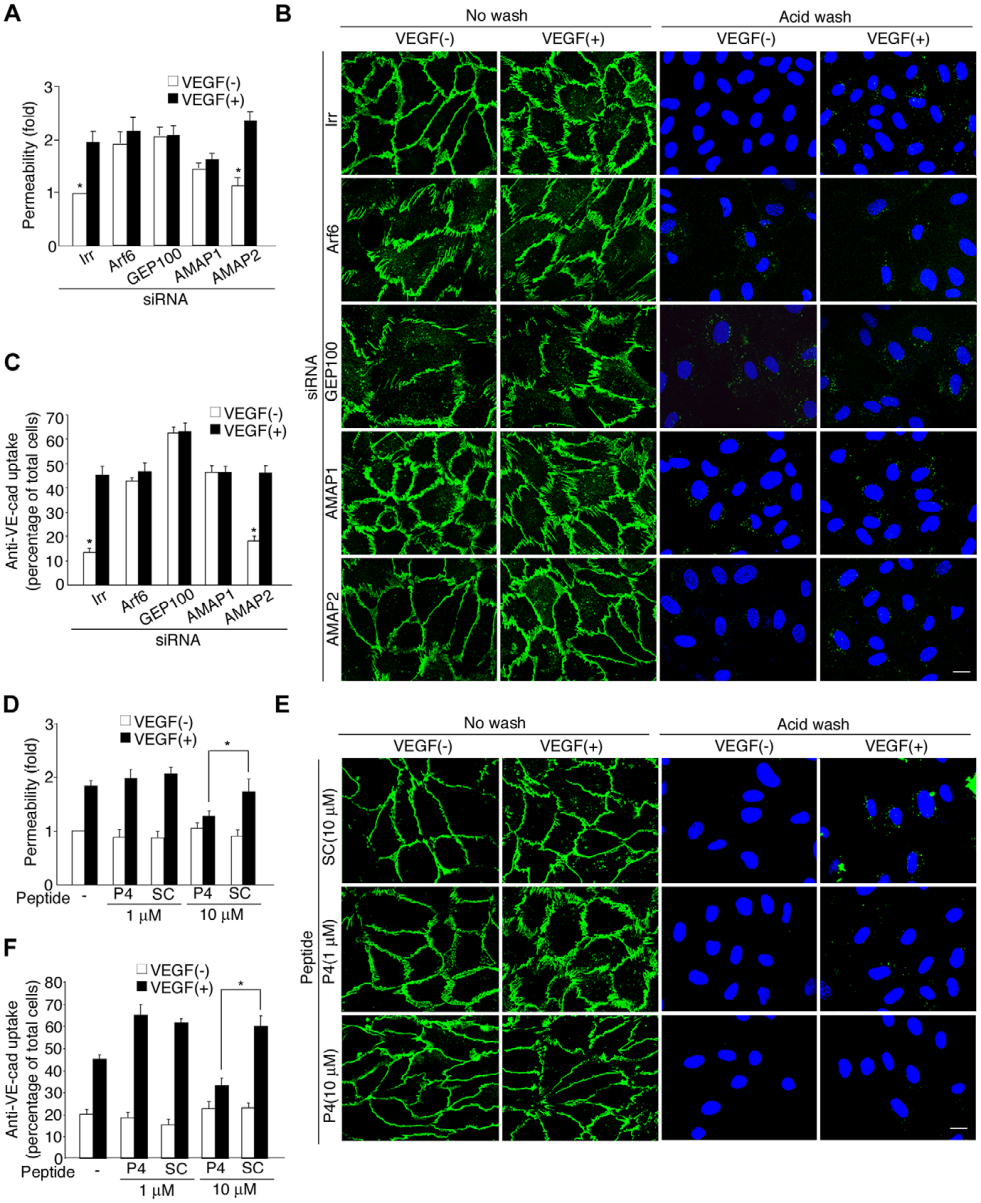
Requirement for the GEP100-Arf6-AMAP1-cortactin pathway in VEGF-induced cell permeability and VE-cadherin endocytosis. HUVECs, treated with siRNAs for Arf6, GEP100, AMAP1 or an irrelevant sequence (Irr) (**A**–**C**), or by P4-TAT (P4) or SC peptide (**D**–**F**), were subjected to a permeability assay by measuring paracellular movement of FITC-dextran (Mr 40 kDa) (**A**, **D**), and to a VE-cadherin uptake assay by tracking endocytosis of cell surface VE-cadherin molecules (**B**, **C**, **E**, **F**). In **A**-**C**, cells were pretreated with siRNAs for 36 h before plating. In **D**–**F**, TAT peptides were added to the confluent culture and incubated for 30 min prior to analysis. VE-cad, green; nuclei, blue. The scale bars represent 10 µm. Error bars show mean ± s.e.m., n = 3. *P<0.05.

We then investigated whether the GEP100-Arf6-AMAP1-cortactin pathway is also involved in endothelial permeability and VE-cadherin endocytosis. VEGF enhances the permeability of intact HUVECs by two fold, measured by paracellular movement of dextran molecules [19], [35] (also see Figure 6A). We then examined the effects of siRNAs for GEP100, Arf6 and AMAP1 on the permeability. For siRNAs to be effective in this assay, siRNA treatment must be started 36 h before the cells are re-plated onto chambers to form confluent monolayers. We found that HUVECs, treated with these siRNAs already exhibit high levels of permeability even without VEGF, and do not respond to VEGF stimulation to change their permeability, (Figure 6A). We next measured the rates of endocytosis of VE-cadherin, from the plasma membrane into the cytoplasm. VEGF accelerates VE-cadherin endocytosis by several folds in intact HUVECs [19] (see Figure 6B and 6C). As in the case of permeability, HUVECs treated with GEP100, Arf6 or AMAP1 siRNAs, all exhibited high rates of the VE-cadherin uptake even without VEGF, and did not respond to VEGF (Figure 6B and 6C). Intact HUVECs exhibit VE-cadherin-based cell-cell junctions with high integrity, while VEGF stimulation evokes their irregular, disorganized morphology [19] (also see Figure 6B). We found that VE-cadherin-based cell-cell junctions become disorganized even without VEGF stimulation, when cells are treated with these siRNAs (Figure 6B). In these experiments, irrelevant and AMAP2 siRNAs were used as negative controls. These results suggest that loss of these proteins disturbs cells to form their intact cell-cell adhesions, and cells with such disorganized cell-cell adhesions are no longer sensitive to VEGF regulation.

We then tested P4-TAT. Unlike the siRNA-treatments described above, P4-TAT can be added directly to cell cultures that are confluent and already form normal, intact cell-cell adhesions. We found that addition of 10 µM P4-TAT to the confluent culture blocks the VEGF-mediated enhancement of the cell permeability, while it does not affect the permeability in the absence of VEGF (Figure 6D). Likewise, P4-TAT blocked VEGF-induced endocytosis of VE-cadherin, while it did not cause internalization of VE-cadherin in cells cultured in the absence of VEGF (Figure 6E and 6F). VEGF-induced morphological changes of the cell-cell junctions were also blocked by P4-TAT (Figure 6E). These effects of P4-TAT were dose-dependent, and the control SC peptide did not exhibit such inhibitory effects (Figure 6D–6F). Taken together, we conclude that the GEP100-Arf6-AMAP1-cortactin pathway is essential for the VEGF regulation of endothelial cell permeability and VE-cadherin endocytosis. Our results also suggest that components of this pathway may essentially be involved in the formation of intact endothelial cell-cell adhesions, as the cell culture medium already contains a low concentration of VEGF.

## Discussion

Angiogenesis and cancer invasion share several common properties [36]. We have shown previously that the GEP100-Arf6-AMAP1-cortactin pathway is used for invasion and metastasis of many breast cancer cells; and in this paper, we show that this pathway is also used in angiogenesis, including breast cancer-induced angiogenesis and choroidal neovascularization. Overexpression of Arf6 and AMAP1 proteins is necessary for the efficient functioning of this pathway in invasion and metastasis [11], [12]. Both Arf6 and AMAP1 are also expressed at high levels in endothelial cells, as seen with highly invasive breast cancer cells. Moreover, this pathway is activated by VEGFR2 in endothelial cells and by EGFR in breast cancer cells [15], both of which are receptor tyrosine kinases.

The GEP100-Arf6-AMAP1-cortactin pathway may at least be involved in the sprouting process of angiogenesis, because remodeling of the VE-cadherin-based cell-cell junctions as well as cell migration/tubular network formation activities are all required in this process. On the other hand, controlling endothelial permeability should not necessarily accompany the sprouting of new tubules, as well as cell migration. We do not know whether the GEP100-Arf6-AMAP1-cortactin pathway can simply regulate endothelial permeability, or VE-cadherin endocytosis, without inducing cell migration or tubular network formation activities (also see below).

VEGF signaling is linked to the activation of Src tyrosine kinases [37], [38]. Our results indicate that VEGFR2 utilizes phospho-Tyr951 to bind to GEP100. Interestingly, VEGFR2 also utilizes phospho-Tyr951 to activate Src kinases, which is mediated by the binding of VRAP to phospho-Tyr951 [39]. VEGFR2-activated Src may then activate the Vav2-Rac-PAK pathway, in which PAK is proposed to phosphorylate VE-cadherin and recruit β-arrestin 2 to induce endocytosis of the phosphorylated VE-cadherin [19]. This pathway was described to be linked to vascular permeability. However, engagement of VRAP, and the activation of Src as well, has also been shown to activate endothelial cell migration [39]. Therefore, like mentioned above, it still remains to be elucidated as to how VEGFR2 signaling pathways, employing Src kinases, can be controlled in order to simply enhance vascular permeability. It also remains to be investigated as to how VEGFR2 chooses to act via GEP100 or VRAP, or both.

It has been reported that ARNO is also responsible for activating Arf GTPases in VEGF signaling [8], [24]. Among the ArfGEFs, HUVECs express GEP100, Cytohesin1, ARNO, BIG2, GBF1 and KIAA0522 at high levels, and Cytohesin3, Cytohesin4, EFA6C and EFA6D at low levels (Figure S6). Several of these, including GEP100 and ARNO, can activate Arf6 [23]. On the other hand, ARNO can also activate Arf1 [23], while GEP100 exhibits a high specificity for Arf6 [40]. It is likely that VEGFR2 signaling employs different ArfGEFs, in addition to GEP100, to activate different Arf GTPases, while the precise mechanisms involved in activating ArfGEFs other than GEP100 largely remain to be clarified.

Several angiogenic inhibitors are already in clinical use, including those targeting VEGFs and VEGFR2 [41], [42]. However, inhibition of all VEGF signaling pathways often exhibits side effects of cardiotoxicity, such as haemorrhage and hypertension [43]. We show that silencing GEP100 does not block other signaling pathways of VEGFR2, such as activating Erk and Akt. Silencing of Arf6, GEP100, and AMAP1 neither affected cell viability. Moreover, tumors that are initially sensitive to VEGF blockage may often develop resistance [42], [44], [45], and hence identification of additional targets is necessary [46]. On the other hand, it is suggested that blockage of tumor angiogenesis, and even disrupting preexisting vascular vessels may have a limitation in killing the tumor cells located at the peripheral rim of tumors [47]. Since the GEP100-Arf6-AMAP1 pathway is common in pathologic angiogenesis and invasion/metastasis of many breast cancers, we propose that components of the GEP100-Arf6-AMAP1-cortactin pathway provide novel molecular targets for treatment of malignant cancers, as well as other diseases characterized by hyper-angiogenesis. Moreover, in both angiogenesis and cancer invasion/metastasis, AMAP1 functions by forming a complex with cortactin. The interface structure of this binding is very unusual [13], and would also be an excellent novel target to inhibit these malignant diseases. Lastly, it is also interesting to examine whether the GEP100-Arf6-AMAP1 pathway is essential also for the cancer neo-vascularization [48], since this pathway may be integral for the generation of cancer stem-like cells [49].

## Supporting Information

Figure S1 **PH domain from GEP100 associates with ligand-activated VEGFR2.** *In vitro* coprecipitation of VEGFR2-V5 with the GST-fused PH domain of GEP100, ARNO or phospholipase Cδ (PLCδ), expressed in Cos-7 cells and analysed by glutathione-beads pulldown and anti-V5 immunoblot. VEGF (10 ng ml^−1^) treatment was for 1 min. Total, total cell lysates (20 µg). GST-fusion proteins were visualized by Ponceau S.
(TIF)

Figure S2 **Tyr951-phosphorylated VEGFR2 was co-immunoprecipitated with GEP100 upon VEGF stimulation.** Phosphorylation of Tyr951 (pY951), Tyr996 (pY996), Tyr1054/1059 (pY1054/1059), Tyr1175 (pY1175), and Tyr1214 (pY1214) of VEGFR2 and its coprecipitation with GEP100 in HUVEC cells, analysed using phosphotyrosine-specific antibodies and anti-GEP100 immunoprecipitation. VEGF (10 ng ml^−1^) treatment was for 1 min. Total, total cell lysates (20 µg).
(TIF)

Figure S3 **Interaction of the PH domain of GEP100 with the pY951 peptide in a dot-blot assay.** Peptides were spotted onto a nitrocellulose membrane (3, 1, and 0.3 µg spot^−1^), and incubated with 5 µg ml^−1^ of GST-PH proteins derived from GEP100 or GST after the membrane was blocked with 5% bovine serum albumin. After washing, GST proteins retained on the membrane were visualized using an anti-GST antibody (left and middle panels). Coomassie brilliant blue (CBB) staining of the membrane is also shown in the right panel. These assays were performed at least two times and representative figures are shown.
(TIF)

Figure S4 **siRNA-mediated knockdown of the expression of either Arf6, GEP100, AMAP1, cortactin or AMAP2 in HUVECs.** Cells were transfected with siRNA duplexes against each indicated molecule, or with irrelevant sequences (Irr), and analysed for expression of the indicated proteins by immunoblotting of cell lysates using the appropriate antibody, as indicated.
(TIF)

Figure S5 **Phosphorylation of Tyr951 (pY951) and tyrosine (pY) of VEGFR2 upon VEGF stimulation of HUVECs.** HUVECs were cultured in low serum (0.5% FCS) medium for 16 h before stimulation. 10 ng ml^−1^ VEGF was used for stimulation for the indicated times, while controls included cells without stimulation (0 min). Total, total cell lysates (20 µg).
(TIF)

Figure S6 **Expression of ArfGEF mRNAs in HUVECs.** Expression of ArfGEF mRNAs was analysed by RT-PCR, coupled with agarose gel electrophoresis. Two ng of cDNA corresponding to each indicated ArfGEF was used as positive controls (PC). NC, without template cDNAs.
(TIF)

Materials and Methods S1 GST-GGA pulldown, immunoprecipitation, and immunoblotting.
(DOCX)
