# Supplementary figures and images for: GEP100-Arf6-AMAP1-Cortactin Pathway Frequently Used in Cancer Invasion Is Activated by VEGFR2 to Promote Angiogenesis
Source: PLoS One. 2011 Aug 15;6(8):e23359. doi: 10.1371/journal.pone.0023359 (PMC3156124; doi:10.1371/journal.pone.0023359)

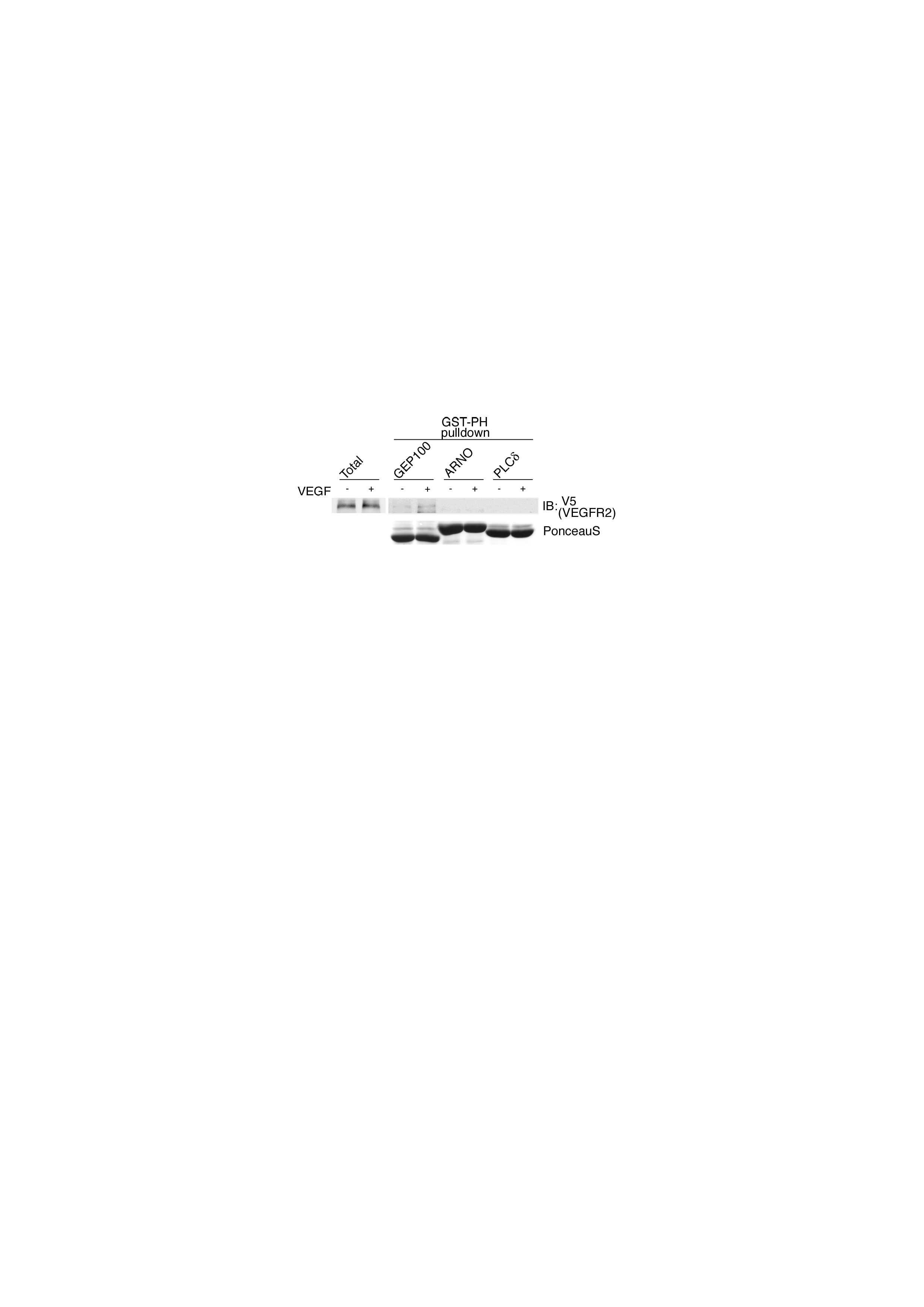

Supplement: Figure S1 — PH domain from GEP100 associates with ligand-activated VEGFR2. In vitro coprecipitation of VEGFR2-V5 with the GST-fused PH domain of GEP100, ARNO or phospholipase Cδ (PLCδ), expressed in Cos-7 cells and analysed by glutathione-beads pulldown and anti-V5 immunoblot. VEGF (10 ng ml−1) treatment was for 1 min. Total, total cell lysates (20 µg). GST-fusion proteins were visualized by Ponceau S. (TIF) [file pone.0023359.s001.tif]

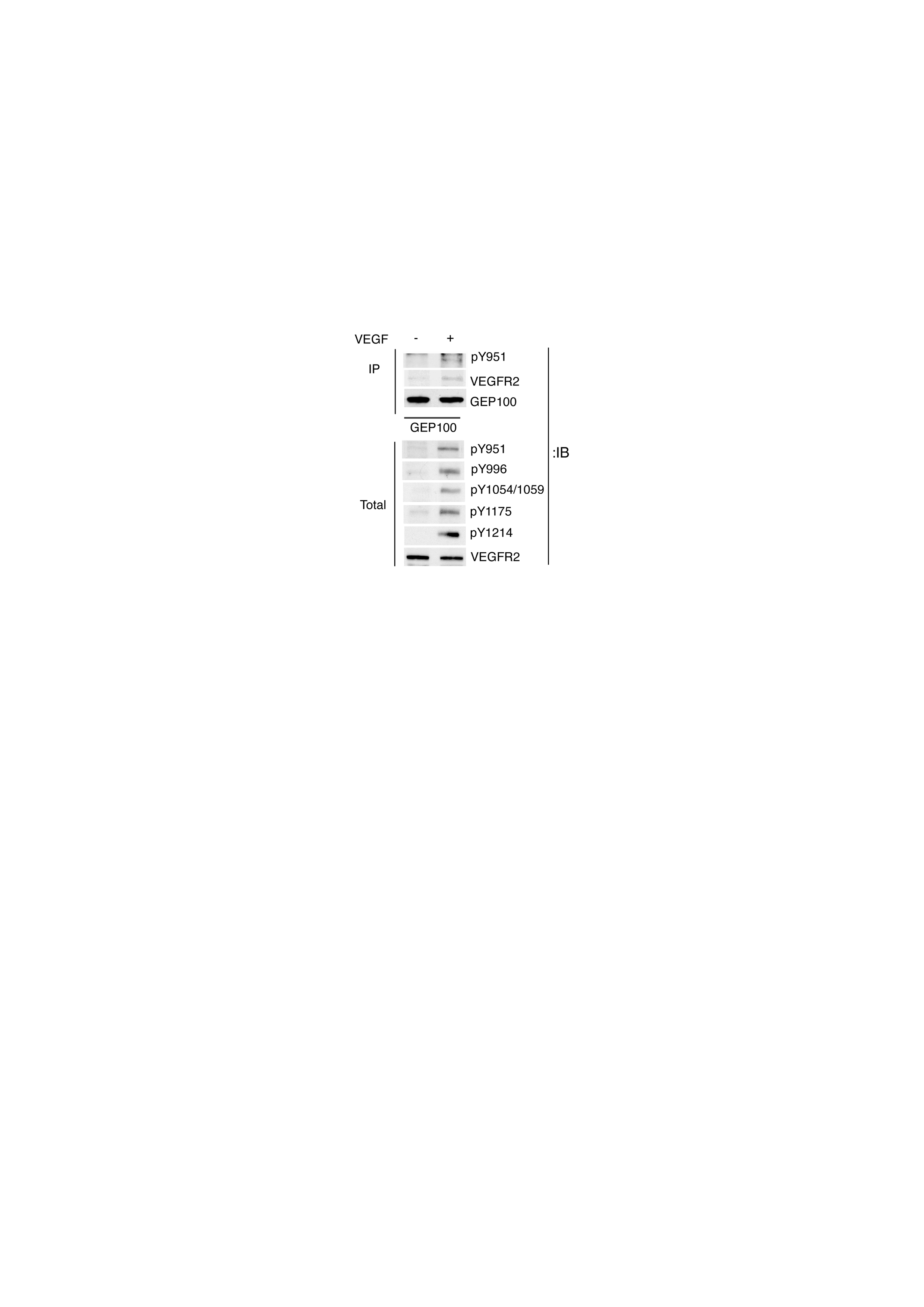

Supplement: Figure S2 — Tyr951-phosphorylated VEGFR2 was co-immunoprecipitated with GEP100 upon VEGF stimulation. Phosphorylation of Tyr951 (pY951), Tyr996 (pY996), Tyr1054/1059 (pY1054/1059), Tyr1175 (pY1175), and Tyr1214 (pY1214) of VEGFR2 and its coprecipitation with GEP100 in HUVEC cells, analysed using phosphotyrosine-specific antibodies and anti-GEP100 immunoprecipitation. VEGF (10 ng ml−1) treatment was for 1 min. Total, total cell lysates (20 µg). (TIF) [file pone.0023359.s002.tif]

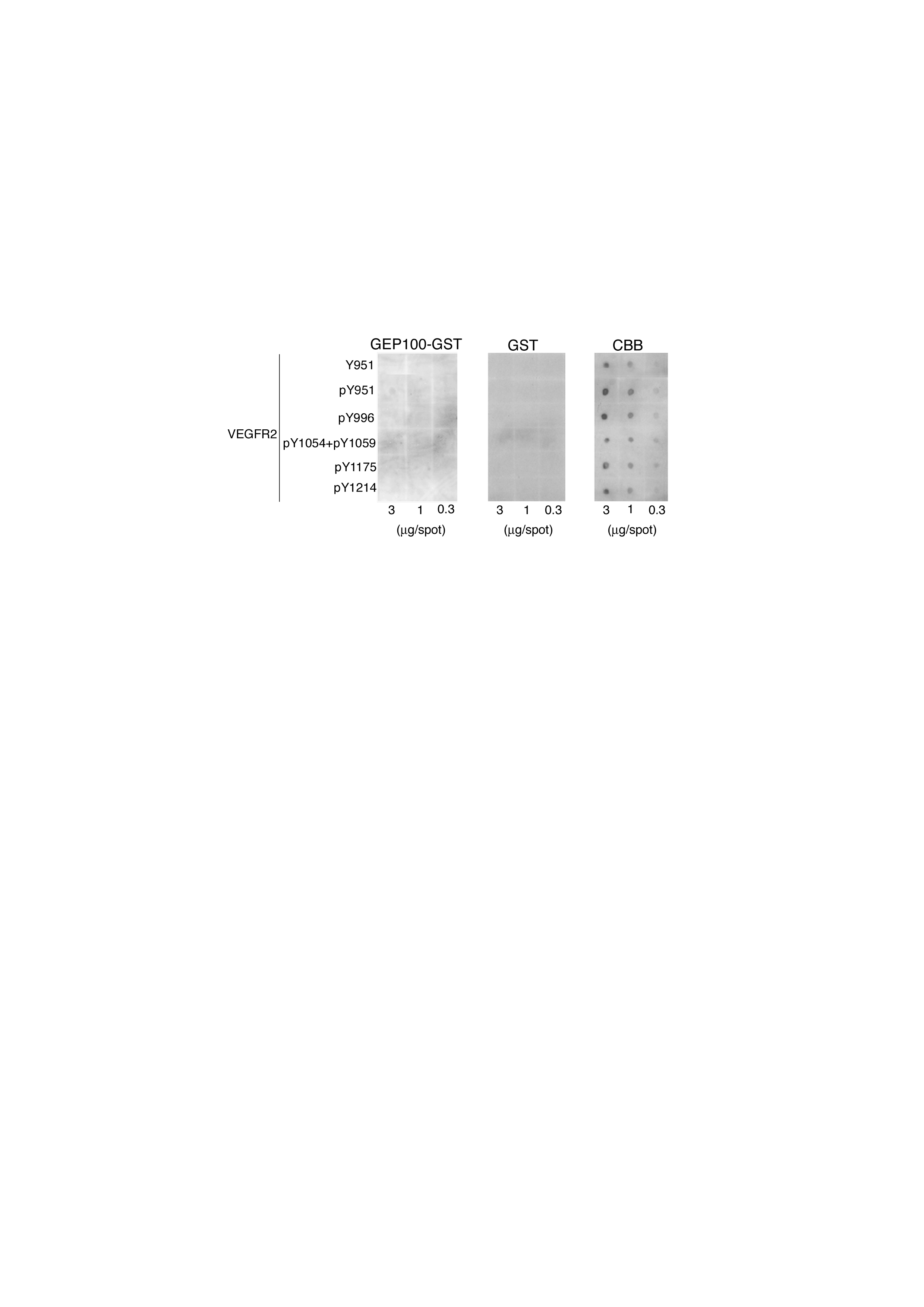

Supplement: Figure S3 — Interaction of the PH domain of GEP100 with the pY951 peptide in a dot-blot assay. Peptides were spotted onto a nitrocellulose membrane (3, 1, and 0.3 µg spot−1), and incubated with 5 µg ml−1 of GST-PH proteins derived from GEP100 or GST after the membrane was blocked with 5% bovine serum albumin. After washing, GST proteins retained on the membrane were visualized using an anti-GST antibody (left and middle panels). Coomassie brilliant blue (CBB) staining of the membrane is also shown in the right panel. These assays were performed at least two times and representative figures are shown. (TIF) [file pone.0023359.s003.tif]

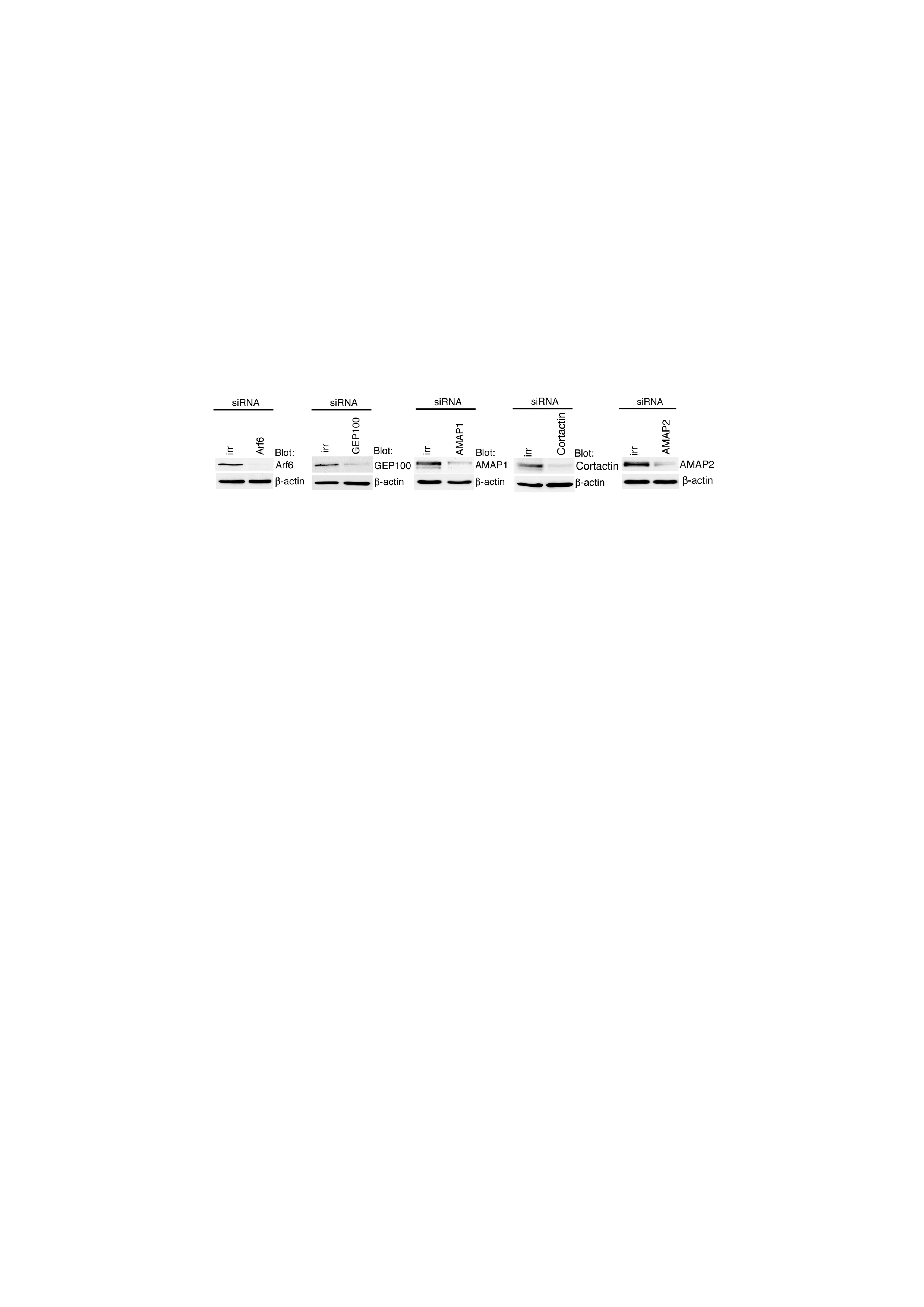

Supplement: Figure S4 — siRNA-mediated knockdown of the expression of either Arf6, GEP100, AMAP1, cortactin or AMAP2 in HUVECs. Cells were transfected with siRNA duplexes against each indicated molecule, or with irrelevant sequences (Irr), and analysed for expression of the indicated proteins by immunoblotting of cell lysates using the appropriate antibody, as indicated. (TIF) [file pone.0023359.s004.tif]

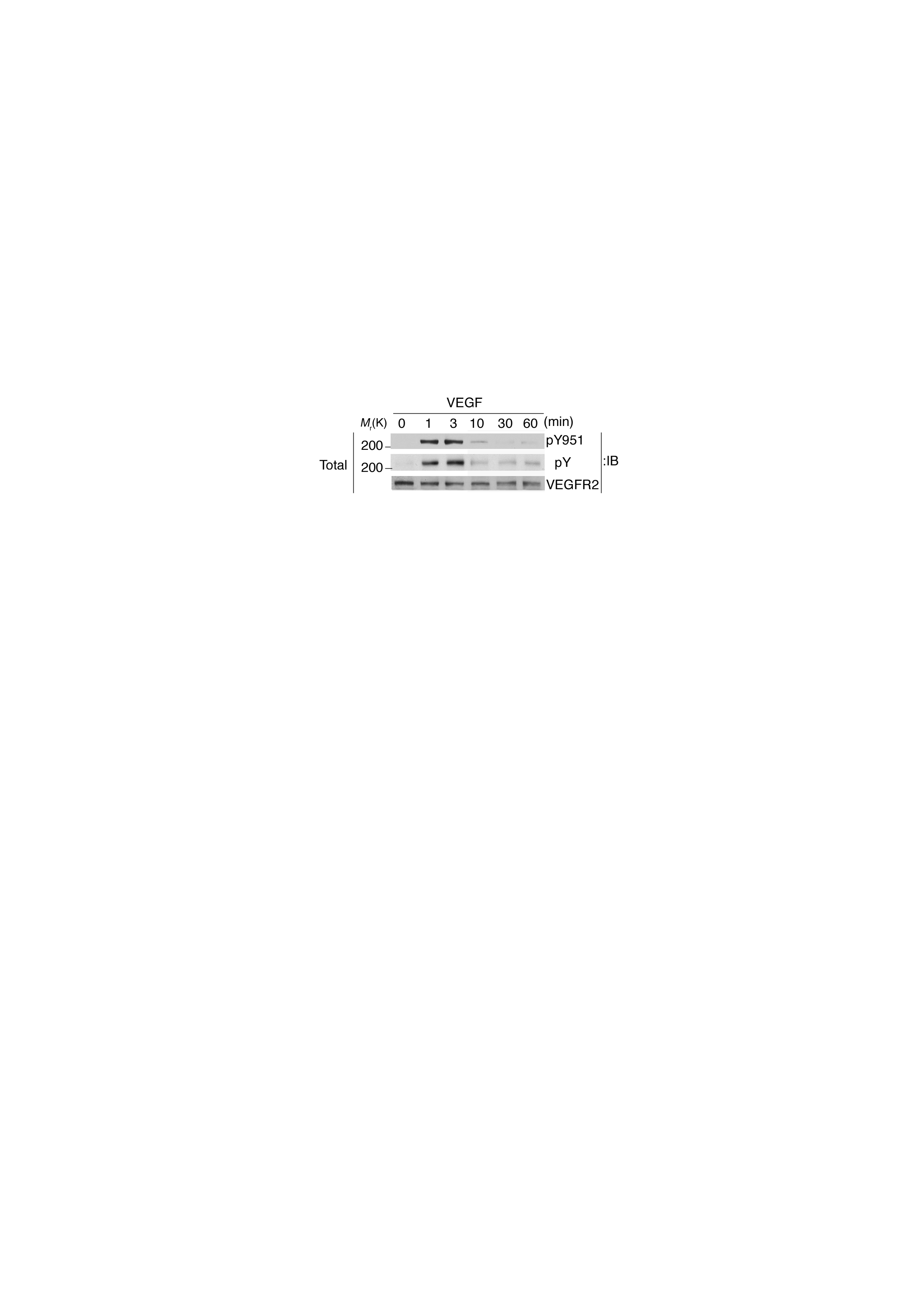

Supplement: Figure S5 — Phosphorylation of Tyr951 (pY951) and tyrosine (pY) of VEGFR2 upon VEGF stimulation of HUVECs. HUVECs were cultured in low serum (0.5% FCS) medium for 16 h before stimulation. 10 ng ml−1 VEGF was used for stimulation for the indicated times, while controls included cells without stimulation (0 min). Total, total cell lysates (20 µg). (TIF) [file pone.0023359.s005.tif]

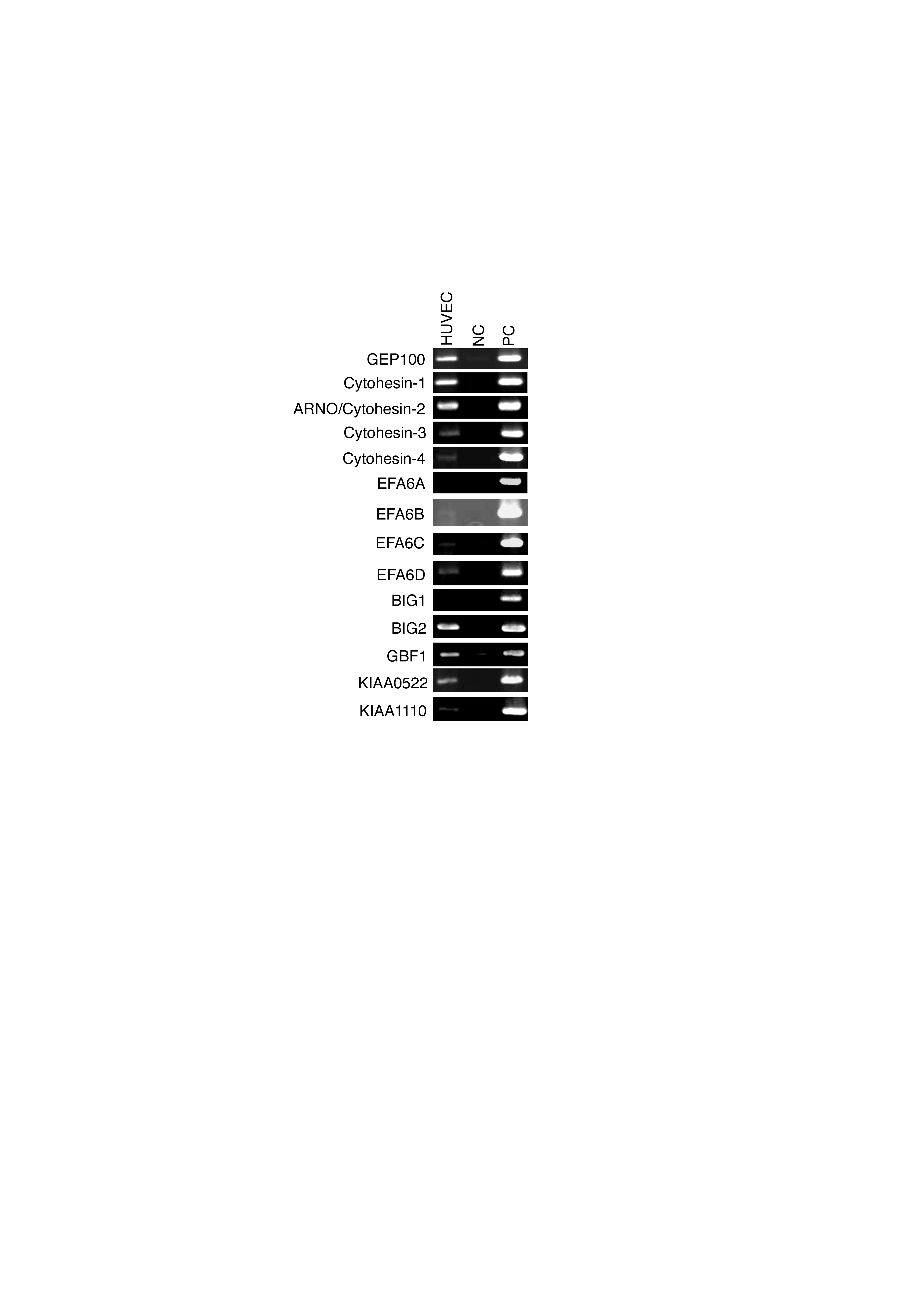

Supplement: Figure S6 — Expression of ArfGEF mRNAs in HUVECs. Expression of ArfGEF mRNAs was analysed by RT-PCR, coupled with agarose gel electrophoresis. Two ng of cDNA corresponding to each indicated ArfGEF was used as positive controls (PC). NC, without template cDNAs. (TIF) [file pone.0023359.s006.tif]
