# Supplementary material for: GEP100-Arf6-AMAP1-Cortactin Pathway Frequently Used in Cancer Invasion Is Activated by VEGFR2 to Promote Angiogenesis
Source: PLoS One. 2011 Aug 15;6(8):e23359. doi: 10.1371/journal.pone.0023359 (PMC3156124; doi:10.1371/journal.pone.0023359)
Supplement: Materials and Methods S1 — GST-GGA pulldown, immunoprecipitation, and immunoblotting. (DOCX) [file pone.0023359.s007.docx]

**Supporting Materials and Methods**

**GST-GGA pulldown, immunoprecipitation, and immunoblotting**

Arf6 activities were measured using GST-GGA, as previously described [1], by using 300 μg of cell lysates in each assay.

For coprecipitation assays of GEP100 with VEGFR2, cells were lysed in RIPA buffer (1% Nonidet P-40, 1% deoxycholate, 0.1% SDS, 20 mM Tris-HCl (pH 7.4), 150 mM NaCl, 5 mM EDTA, 1 mM Na_3_VO_4_, 1 mM phenylmethylsulfonyl fluoride, 5 μg ml^-1^ aprotinin, 2 μg ml^-1^ leupeptin, and 3 μg ml^-1^ pepstatin A). 1 mg of cell lysates were then incubated with an anti-GEP100 polyclonal antibody or an anti-HA antibody (for HA-GEP100), coupled to Protein A-Sepharose beads.

Immunoblotting analysis, coupled with SDS-PAGE, was performed as described previously [2].

Antibodies and chemicals

Rabbit polyclonal antibodies against GEP100, AMAP1 and AMAP2 were described previously [1, 3]. Other antibodies were purchased from commercial sources: mouse monoclonal antibodies against V5-tag (Invitrogen), HA-tag (BD Biosciences), VEGFR2 (Santa Cruz), anti-phosphotyrosine (pY) (4G10 clone; UBI), β-actin (UBI), Arf6 (Santa Cruz), Thr-202/Tyr204 phosphorylated Erk (Cell Signaling), cortactin (UBI), VE-cadherin (BV6 clone; Millipore) and CD31 (BD Pharmingen). Rabbit polyclonal antibodies against VEGFR2 phosphorylated at Tyr951, Tyr996, Tyr1175 and Tyr1214 (Cell Signaling), and Tyr1054/1059 (Biosource); Erk, Akt and its Ser-473 phosphorylated form (Cell Signaling). Donkey antibodies against rabbit and mouse IgGs, conjugated with horseradish peroxidase, were from Jackson ImmunoResearch Laboratories. VEGF-A was purchased from PeproTech. All other chemical reagents were purchased from Sigma-Aldrich and Wako, unless otherwise described.

**cDNAs**

cDNAs encoding GEP100, Arf6, PLCδ PH and GST-GGA3 were as described previously [1]. cDNA encoding VEGFR2 (pEF-DEST51/VEGFR2 V5 for V5-tagged VEGFR2) was a gift from M. Kitakaze (National Cadiovascular Center, Osaka, Japan). For construction of YF mutants of VEGFR2, point mutations were introduced using PCR-mediated mutagenesis. Oligonucleotides used were 5’-CAAGGGAAAGACTTCGTTGGAGCAATCCCTGTG-3’, 5’-GATGGCAAAGACTTCATTGTTCTTCCGATATCAGAG-3’ and 5’-CCCAAATTCCATTTCGACAACACAGCAGGAATC-3’, which mutate tyrosines 951, 1175 and 1214 into phenylalanines, respectively. PCR products were ligated into the *BstXI-XbaI* site of pEF-DEST51/VEGFR2 V5. Mutations were verified by sequencing.

**siRNA**

HUVECs were trypsinized, washed with Hepes buffered saline solution, and resuspended (10^6^ cells in 100 ml) in a HUVEC solution (Amaxa Biosystems) containing 1 nM siRNA duplexes, and were electroporated using a Nucleofector (Amaxa Biosystems), according to the manufacturer’s instructions. Cells were then plated onto culture dishes and incubated for 48 h in EGM2 with 2% FCS prior to analysis, unless otherwise described.

Nucleotide sequences used were: for *GEP100,* 5’-AAGTGAAATCACTGGCCGAGT-3’; for *Arf6,* 5’-AA GCACCGCATTATCAATGACCG-3’; for *AMAP1,* 5’-AA GACCTGACAAAAGCCATTA-3’; for *cortactin,* 5’-AA GGACAAAGTGGATAAGAGC-3’; and for *AMAP2,* 5’-AA AGAGGACTCCCAAATTCGT-3'. An siRNA duplex with an irrelevant sequence (5'-GCGCGCUUUGUAGGAUUCG-3') was purchased from Dharmacon.

**Transfections**

cDNA transfections were performed using Nucleofector for HUVECs, and Polyfect (Qiagen) for Cos-7 cells, according to the manufacturer’s instructions.

5x10^5^ Cos-7 cells were co-transfected with 3 μg of pcDNA HA-GEP100 and 3 μg of pEF-DEST51/VEGFR2 V5 or its YF mutants for coprecipitation assays; and with 0.3 μg of pcDNA Arf6-HA, 3 μg pcDNA HA-GEP100 and 4 μg of pEF-DEST51/VEGFR2 V5 or its YF mutants for Arf6 activity assays. After incubation for 24 h in DMEM with 10% FCS, cells were starved for serum for 16 h, and then stimulated with or without 10 ng ml^-1^ VEGF for 1 min, prior to analyses.

**Tubular formation**

Analysis of the formation of vascular-like structures by HUVECs was performed according to a method reported previously [4]. Briefly, cells were plated onto collagen-coated plates at 1.5x10^5^ cells/well in EGM2 with 0.5% FCS. Sixteen h later, cells were overlayed with collagen gel containing 10 ng ml^-1^ VEGF, and further incubated for 48 h. Tubular formation was then observed using an inverted phase contrast microscope (Olympus). Images were captured using AxioVision (Carl Zeiss).

**Chemotactic transwell cell migration**

Chemotactic transwell cell migration activity was measured using modified Boyden chambers, as described previously [5]. Briefly, 1x10^4^ HUVECs, starved for 16 h in EGM2 with 0.5% FCS, were added to the transwell with a 0.1% gelatin-coated insert (6.4 mm diameter, 3.0 μm pore size; BD Biosciences), in which lower chambers were filled with EGM2 with 0.5% FCS containing 20 ng ml^-1^ VEGF. After 4 h incubation, cells that transmigrated into the lower side of the chamber were scored.

**Two dimensional cell migration activity**

Two dimensional cell migration activity was measured by a wound healing assay, as described previously [4]. Briefly, HUVECs were plated onto collagen (Type I, 10 μg ml^-1^)-coated plates at 1.5x10^5^ cells/well, and after being starved for 16 h in EGM2 with 0.5% FCS, confluent monolayers of cells were wounded with a 200-μl pipette tip and incubated for 48 h in EGM2 with 0.5% FCS containing 20 ng ml^-1^ VEGF. Photographs were then taken using an inverted phase contrast microscope (Olympus) coupled with Axiovision.

**Immunohistochemical staining**

Immunohistochemistry was performed on 4 μm thick formalin-fixed paraffin-embedded sequential sections. Immunohistochemical staining of CD31, GEP100 or AMAP1 was performed using the standard avidin-biotin-peroxidase complex (ABC) method, as previously described [1]. Briefly, sections were deparaffinized in xylene and dehydrated in a graded series of ethanol, and processed for antigen retrieval by heating in 10 mM sodium citrate (pH 6.0) at 121˚C for 5 min in a pressure cooker. Endogenous peroxidase activity was blocked by 0.3% H_2_O_2_ in methyl alcohol at room temperature for 30 min. After rinsing in PBS and blocking with 1% goat serum in PBS at room temperature for 30 min, the sections were incubated with antibodies against the above proteins in PBS overnight at 4˚C. After rinsing in PBS, the sections were incubated with a biotinylated goat anti-rabbit IgG antibody or a biotinylated horse anti-mouse IgG antibody (Vector Lab) for 40 min and then with peroxidase-conjugated streptavidin (Vector Lab) at room temperature for 50 min. After rinsing in PBS, the coloring reaction was carried out with 0.3 mg ml^-1^ diaminobenzidine and 0.003% H_2_O_2_ in 50 mM Tris-HCl (pH 7.6). Each section was counterstained with hematoxylin.

**Cell permeability**

Permeability was measured by the passage of FITC-dextran (Mr 40 kDa, Molecular Probes), as described [6, 7]. Briefly, 1x10^4^ HUVECs were plated onto 6.5 mm Transwell Collagen-coated 3 μm pore PTFE membrane inserts (Corning Costar) and incubated for 2 days to form confluent monolayers. Then, after incubated with EGM2 without serum for 4 h, FITC-dextran (1 mg ml^-1^) was added onto the cell culture in the presence or absence of VEGF (50 ng ml^-1^) and incubated for a further 30 min. For siRNA treatment, cells were preincubated with RNA duplexes for 36 h, before being plated onto the inserts. TAT peptides were added onto the confluent cell monolayers simultaneously with FITC-dextran. Amounts of fluorescent dextran accumulated within the bottom chambers were measured using a multi-plate fluorescent reader (ARVO). Statistical analysis was performed using ANOVA.

**Immunofluorescent microscopy**

Immunofluorescence staining of cells and acquisition of confocal images were performed using a confocal laser scanning microscope (LSM510; Carl Zeiss), as previously described [2].

**GST-PH binding**

For *in vitro* protein binding assays, 25 μg each of GST-fused PH domains of GEP100 (aa 631-742), ARNO (aa 261-387) or PLCδ (aa 16-128), expressed in bacteria and purified on glutathione-beads, and 1 mg of Cos-7 cell lysates expressing VEGFR2-V5 prepared in RIPA buffer were used. cDNA transfected Cos-7 cells were incubated for 24 h in DMEM with 10% FCS, then starved for serum for 16 h and stimulated with 10 ng ml^-1^ VEGF for 1 min, prior to lysis.

**Viability**

Cell viabilities were measured using a 3-(4,5-dimethylthiazol-2-yl)-5-(3-carboxyphenyl)-2-(4-sulfophenyl)-2H-tetrazolium (MTS) colorimetric assay kit (Promega), according to the manufacturer’s instructions.

**Peptides**

Peptides were synthesized by Sigma Genosys. The sequences of VEGFR2-derived peptides were as follows: peptide pY951, RQGKDYVGAI; pY996, EAPEDLYKDFLT; pY1054+pY1059, RDIYKDPOYVRKG; pY1175, AQQDGKDYIVLPI; and pY1214, DPKFHYDNTAG. Tyrosines in each peptide were phosphorylated.

**RT-PCR**

Total RNA was extracted from cultured cells using TRIzol reagent (Invitrogen) according to the manufacturer’s protocol and reverse-transcribed by M-MLV Reverse transcriptase (Promega) using oligo dT primers at 42 ℃ for 60 min. cDNAs were then subjected to 35 cycles of PCR amplification, as previously described [1].

**Supplementary References**

1. Morishige M, Hashimoto S, Ogawa E, Toda Y, Kotani H, et al*.* (2008) GEP100 links epidermal growth factor receptor signalling to Arf6 activation to induce breast cancer invasion. Nat Cell Biol 10: 85-92.
2. Hashimoto S, Onodera Y, Hashimoto A, Tanaka M, Hamaguchi M, et al*.* (2004) Requirement for Arf6 in breast cancer invasive activities. Pro Natl Acad Sci U S A 101: 6647-6652.
3. Onodera Y, Hashimoto S, Hashimoto A, Morishige M, Mazaki Y, et al. (2005) Expression of AMAP1, an ArfGAP, provides novel targets to inhibit breast cancer invasive activities. EMBO J 24: 963-973.
4. Matsui J, Wakabayashi T, Asada M, Yoshimatsu K, Okada M (2004) Stem cell factor/c-kit signaling promotes the survival, migration, and capillary tube formation of human umbilical vein endothelial cells. J Biol Chem 279: 18600-18607.
5. Ouchi N, Kobayashi H, Kihara S, [Kumada M](http://www.ncbi.nlm.nih.gov/pubmed?term=%22Kumada%20M%22%5BAuthor%5D), [Sato K](http://www.ncbi.nlm.nih.gov/pubmed?term=%22Sato%20K%22%5BAuthor%5D), et al. (2004) Adiponectin stimulates angiogenesis by promoting cross-talk between AMP-activated protein kinase and Akt signaling in endothelial cells. J Biol Chem 279: 1304-1309.
6. Gavard J, Gutkind JS (2006) VEGF controls endothelial-cell permeability by promoting the b-arrestin-dependent endocytosis of VE-cadherin. Nat Cell Biol 8: 1223-1234.
7. Fukuhara S, Sakurai A, Sano H, Yamagishi A, Somekawa S, et al. (2005) Cyclic AMP potentiates vascular endothelial cadherin-mediated cell-cell contact to enhance endothelial barrier function through an Epac-Rap1 signaling pathway. Mol Cell Biol 25: 136-146.
